# Association between weight-adjusted waist index and overactive bladder: a cross-sectional study based on 2009–2018 NHANES

**DOI:** 10.3389/fnut.2024.1423148

**Published:** 2024-09-04

**Authors:** Zeng Hui, Zhu Zewu, Li Yang, Cui Yu

**Affiliations:** ^1^Nursing Department, The Third Xiangya Hospital, Central South University, Changsha, Hunan, China; ^2^Department of Urology, Xiangya Hospital, Central South University, Changsha, Hunan, China

**Keywords:** weight-adjusted waist index, overactive bladder, NHANES, body mass index — BMI, waist circumference

## Abstract

**Background:**

The weight-adjusted waist index (WWI) is a novel obesity indicator that appears to outperform the body mass index (BMI) and waist circumference (WC) in assessing both overweight and obesity. Studies have demonstrated the relationship between obesity and overactive bladder (OAB). The purpose of this study is to examine the correlation between WWI and OAB.

**Methods:**

This research utilizes data from the National Health and Nutrition Examination Survey (NHANES) collected between 2009 and 2018. Each participant’s WWI was calculated as their WC in centimeters by the square root of weight in kilograms. The Overactive Bladder Symptom Score (OABSS) questionnaire is used to determine whether a participant has OAB. Multivariate logistic regression and generalized additive model analysis were employed to investigate the relationship between WWI and OAB. We used smoothing curve fitting to explore non-linear relationships. Additionally, subgroup analysis and interaction tests are conducted.

**Results:**

In this cross-sectional study involving 35,950 subjects, we found that individuals with a higher WWI have a higher risk of OAB (OR = 1.41, 95% CI: 1.02–1.74). Subgroup analysis and interaction testing showed that the relationship between WWI and OAB is consistent across various population characteristics. Smoothing curve fitting reveals a positive non-linear relationship between WWI and OAB. Furthermore, the association between WWI and OAB is stronger than that of other obesity-related indicators.

**Conclusion:**

Weight-adjusted waist index may be able to predict the incidence of OAB and that WWI-based obesity management may help to reduce the risk of OAB.

## 1 Introduction

Overactive bladder (OAB) is a symptom-based definition, characterized by urgency symptoms and often accompanied by frequency and nocturia, with or without urgency urinary incontinence (1, 2). It significantly affects patients’ daily lives and social activities, making it a major health concern. According to epidemiological surveys, the prevalence of OAB in adults is 16.5% in the United States (3). In China, based on the first large-scale epidemiological survey data released by the Chinese Medical Association Urology Branch in June 2010, the overall prevalence of OAB in the population aged 18 and above is 5.9%, and it gradually increases with age, reaching 11.3% in individuals aged 40 and above (4). These data highlight the high prevalence of OAB and warrant the attention of clinical urologists.

The OAB risk factors included obesity, smoking, fluid intake, consumption of carbonated beverages, and diet (5). Recent studies have shown that obesity can increase the pressure on the bladder and surrounding muscles, and excessive accumulation of intra-abdominal fat may lead to the release of excessive inflammatory factors by inflammatory cells, thereby affecting the adipocytes around the bladder and triggering OAB symptoms (6–8). Therefore, a method is needed to assess the amount of abdominal fat tissue and its relationship with OAB. Currently, there are several anthropometric measures, such as body mass index (BMI) and waist circumference (WC), that have been used to measure obesity. The results regarding their association with OAB are still contradictory (9–14). Moreover, BMI does not describe the actual distribution of body fat. Park et al. (15) proposed a new anthropometric measure called weight-adjusted waist circumference index (WWI), which can maximize the gains of WC and minimize the association with BMI. The WWI serves not only to differentiate between adipose and muscular tissues but also to address issues of central obesity that are not directly related to overall body weight. Many studies have shown that there is a positive correlation between WWI and hypertension, diabetes, even all-cause mortality and cardiovascular mortality (16–18). Although WWI is a better predictor of obesity compared to BMI, its relationship with OAB has not been studied before.

To gain a more accurate understanding of the association between obesity and OAB, it is necessary to determine the relationship between obesity assessed by WWI and OAB. Therefore, we used data from the National Health and Nutrition Examination Survey (NHANES) from 2009 to 2018 to investigate the relationship between WWI and OAB.

## 2 Materials and methods

### 2.1 Data source

NHANES is a study conducted by the National Center for Health Statistics (NCHS) in the United States, aimed at assessing the health and nutritional status of the U.S. population. The public has access to all NHANES data through the official website of the CDC: https://www.cdc.gov/nchs/nhanes/. The sample used for NHANES has considerable representativeness due to the use of a stratified multistage probability sampling design in the study. NHANES data is released to the public in two-year cycles.

### 2.2 Study population

**Figure 1:**
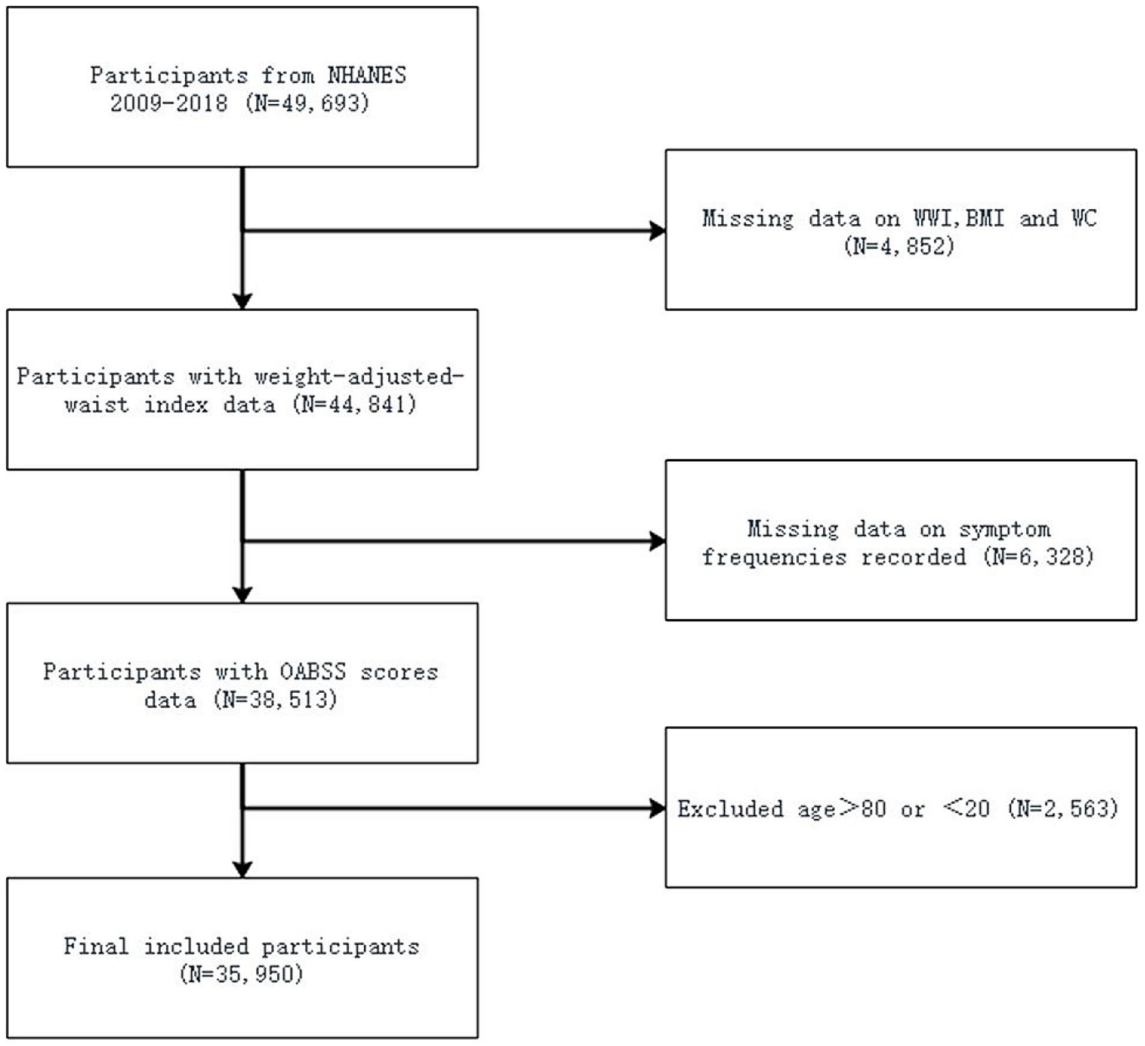
Flowchart of the sample selection from NHANES 2009–2018.

Our analysis involved 10 years (2009–2010, 2011–2012, 2013–2014, 2015–2016, 2017–2018) of NHANES study data, including 49,693 participants. After excluding participants without complete information about UUI, incomplete nocturia frequency data, and without available information about BMI, WC and WWI, a portion of participants remained. Additionally, participants older than 80 years and younger than 20 years were also excluded. Finally, a total of 35,950 participants were included in this study (Figure 1).

### 2.3 Assessment of weight-adjusted-waist index

Weight-adjusted waist index is a body measurement index based on WC and weight, used to assess central obesity. Body measurement data regarding WC and weight were provided by trained health technicians from NHANES. The WWI for each participant was calculated by dividing WC in centimeters by weight in kilograms squared, rounded to two decimal places (WWI = WC/weight^2, with WC in centimeters and weight in kilograms). Higher WWI values indicate increased obesity levels.

### 2.4 Assessment of OAB

Overactive bladder is defined as an overactive voiding reflex characterized by urgency urinary incontinence (UUI) and nocturia (19). All the information obtained is based on questionnaire surveys, which were conducted by trained researchers through face-to-face interviews. UUI was obtained through the following question: “During the past 12 months, have you leaked or lost control of even a small amount of urine with an urge or pressure to urinate and you could not get to the toilet fast enough?” The severity was assessed through the following question: “How frequently does this occur?” In addition, the gravity of nocturia was assessed through the following question: “During the past 30 days, how many times per night did you most typically get up to urinate, from the time you went to bed at night until the time you got up in the morning.” Furthermore, OAB was qualified using the Overactive Bladder Symptom Score (OABSS), as shown in Supplementary Table S1. A participant with a total OABSS score ≥ 3 was considered to have OAB.

### 2.5 Assessment of covariates of interest

In this study, covariates included age, gender, race, education level, marital status, alcohol drinks, smoking history, hypertension, diabetes mellitus, BMI, WC and WWI. The inclusion of these covariates was determined professionally after reviewing existing studies. These covariates were sourced from the demographic, examination, and questionnaire sections of the NHANES database.

### 2.6 Statistical analysis

According to CDC guidelines, all statistical analyses were conducted using appropriate NHANES sampling weights, taking into account the complex sampling design and multiple stages of cluster sampling. Weighted Student’s *t*-tests were used to assess the differences among WWI for continuous variables, and weighted chi-square tests were used for categorical variables. Due to the complex multistage probability sampling design of NHANES, inferential statistical methods were used to represent a nationally representative large sample. Therefore, linear regression analysis was used to summarize continuous variables as means with standard errors, and logistic regression analysis was used to summarize categorical parameters as proportions. To examine the association between WWI and OAB, weighted multivariable regression models were used in three different models. In Model 1, no adjustment was made for covariates. Model 2 adjusted for gender, age, and race. Model 3 adjusted for gender, age, race, education level, marital status, alcohol drinks, smoking history, hypertension, diabetes Mellitus, BMI, and WC. Smooth curve fitting were used to address non-linearity issues. When non-linear associations were observed, segmented regression models were fitted within each interval and threshold effects were calculated. A two-step recursive method was used to further determine the breakpoint (K) that connects the segments, which was based on the model that yields the maximum likelihood. Data extraction, screening, and statistical analysis were conducted using R 4.1.2 (Institute of Statistics and Mathematics, Vienna, Austria). *P* < 0.05 served as the criterion for significance.

## 3 Results

### 3.1 Baseline characteristic

This study included a total of 35,950 participants aged 20–80 years. The population baseline characteristics are shown in Table 1, based on their OAB status. There were significant differences between the groups with and without OAB in terms of age, gender, marital status, alcohol use, diabetes mellitus, hypertension, BMI, WC and WWI. Compared to participants without OAB, individuals with OAB were more likely to be middle-aged or older (40–80 years), female, married, alcohol use, diabetes, hypertension, have a higher BMI, WC and WWI.

**Table 1:** Baseline characteristics of study participants.

Characteristic	Overactive bladder		*P*-value
	No	Yes	
	*N* = 29840	*N* = 6110	
Total patients			
Age (years, %)			<0.01
20–40	40.32	16.26	
40–60	36.51	39.15	
60–80	23.17	44.59	
Gender (%)			<0.01
Male	43.27	25.74	
Female	56.73	74.26	
Race (%)			0.13
White	63.21	61.40	
Black	24.35	23.47	
Other	12.44	15.13	
Education level (%)			0.21
Less than high school	12.63	17.22	
High school	24.16	27.05	
More than high school	63.21	65.73	
Marital status (%)			<0.01
Married/living with partner	65.18	78.63	
Single/divorced/widowed	34.82	21.37	
Alcohol user (%)			<0.01
Yes	61.33	75.35	
No	38.67	24.65	
Smoke (%)			0.08
Yes	29.64	36.78	
No	70.36	63.22	
Hypertension (%)			<0.01
Yes	28.67	34.43	
No	71.33	65.57	
Diabetes Mellitus (%)			<0.01
Yes	10.35	19.23	
No	89.65	80.77	
BMI (%)			<0.01
<25	36.84	31.28	
≥25	63.16	68.72	
Waist circumference (cm)	93.25±12.31	106.38±9.23	<0.01
Weight-adjusted-waist index	9.23±1.41	11.37±0.21	<0.01

### 3.2 The association between WWI and OAB

Table 2 displays the association between WWI and OAB. Our study findings indicate that higher WWI is associated with an increased risk of OAB. Both crude model and the minimally/fully adjusted model showed evidence of a positive correlation between WWI and OAB. In the fully adjusted model, the independent effect between the two was (Model 3: OR = 1.41, 95% CI: 1.02–1.74), indicating that participants with a unit higher WWI had a 41% increased risk of OAB. After categorizing WWI into tertiles, this association remained statistically significant. Compared to participants in the lowest tertile, those in the highest tertile had a 126% increased risk (OR = 2.26, 95%CI: 1.32–2.52; *p* < 0.01).

**Table 2:** Associations between WWI and the risk of OAB.

Characteristics	Model 1	Model 2	Model 3
WWI (continuous)	1.52 (1.16, 1.73) *P* < 0.01	1.46 (1.12, 1.63) *P* < 0.01	1.41 (1.02, 1.74) *P* = 0.03
Tertile1	Reference	Reference	Reference
Tertile2	1.94 (1.45, 2.33) *P* < 0.01	1.72 (1.41, 2.03) *P* < 0.01	1.65 (1.12, 1.94) *P* < 0.01
Tertile3	2.85 (1.95, 3.25) *P* < 0.01	2.54 (1.85, 2.94) *P* < 0.01	2.26 (1.32, 2.52) *P* < 0.01
*P* for trend	*P* < 0.01	*P* < 0.01	*P* < 0.01

### 3.3 Subgroup analysis

Subgroup analysis was conducted to assess the stability of the relationship between WWI and OAB across different population backgrounds. The results showed that all factors had no significant impact on the relationship between WWI and OAB. As shown in Table 3, the associations between WWI and the risk of OAB remained consistent in different subgroups by age, gender, race, education lever, marital status, smoking, alcohol drinks, hypertension, and diabetes mellitus (all *P* for interaction > 0.05).

**Table 3:** Subgroups analyses of the effect of WWI on OAB.

Subgroups	OR (95%CI), *P*-value	*P* for interaction
Age (years)		0.07
20–40	1.24 (0.85–1.43) 0.24	
40–60	1.32 (1.03–1.64) 0.02	
60–80	1.41 (1.12–1.54) <0.01	
Gender		0.39
Male	1.22 (0.93–1.42) 0.07	
Female	1.45 (1.27–1.74) <0.01	
Race		0.65
White	2.13 (0.75–2.53) 0.37	
Black	1.76 (0.85–1.86) 0.56	
Other	1.64 (1.33–2.47) <0.01	
Education level		0.26
Less than high school	0.91 (0.51–1.43) 0.72	
High school	1.68 (1.32–2.41) <0.01	
More than high school	1.49 (1.13–2.07) <0.01	
Marital status (%)		0.18
Married/living with partner	1.29 (0.81–1.67) 0.52	
Single/divorced/widowed	1.36 (1.12–1.67) <0.01	
Alcohol user (%)		0.14
Yes	2.35 (1.42–2.96) <0.01	
No	1.26 (0.72–2.04) 0.67	
Smoke (%)		0.64
Yes	1.25 (1.10–1.89) <0.01	
No	0.97 (0.84–1.42) 0.31	
Hypertension		0.27
Yes	1.65 (1.32–1.97) <0.01	
No	1.32 (0.84–1.67) 0.26	
Diabetes Mellitus		0.35
Yes	1.51 (1.23–1.80) <0.01	
No	1.29 (0.85–1.78) 0.32	

### 3.4 Smoothed curve fitting and threshold effect analysis

**Figure 2:**
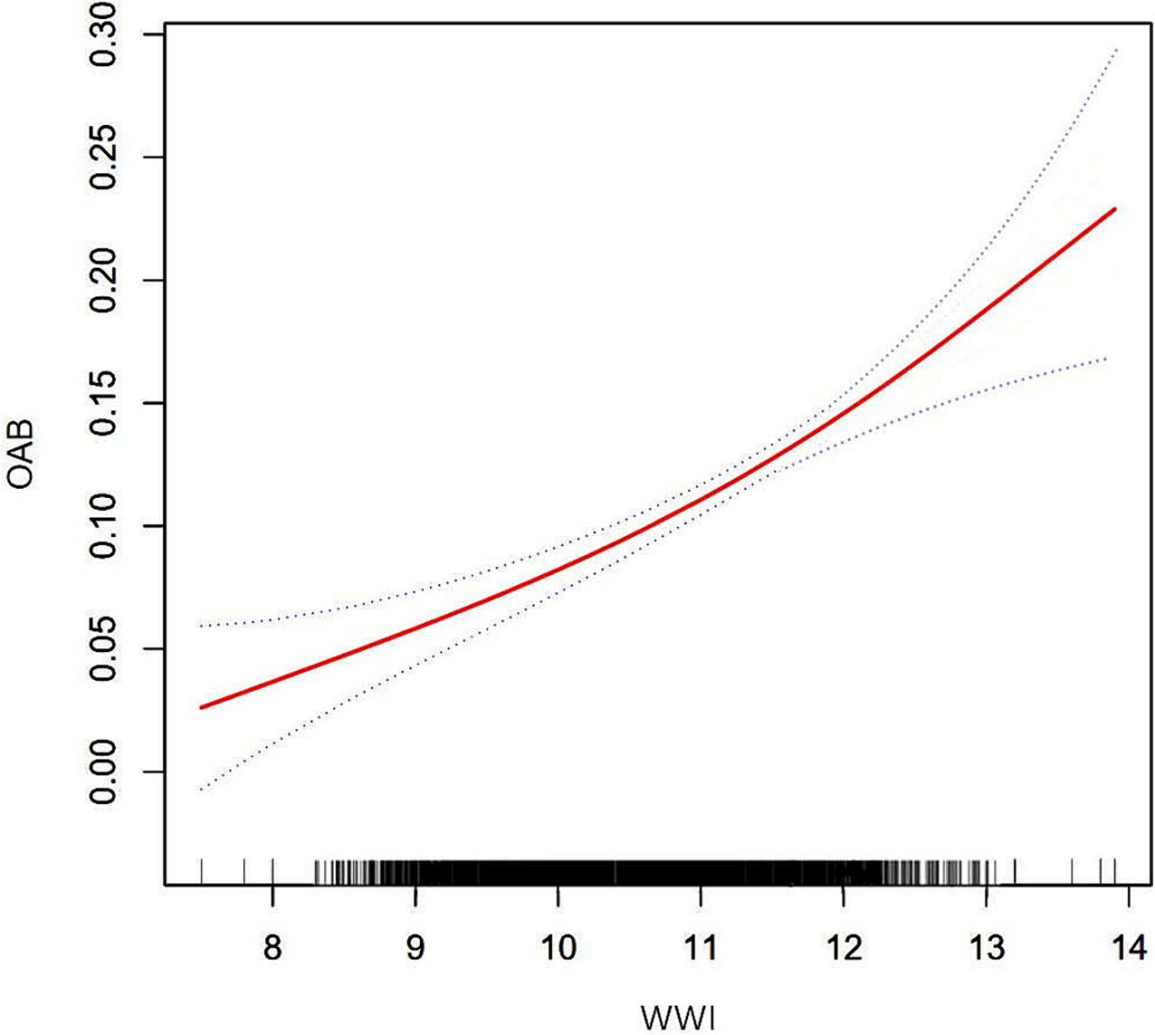
Smooth curve fitting for OAB and WWI.

To assess whether the positive correlation between WWI and OAB was non-linear, we conducted further smooth curve fitting. The results confirmed that participants with higher WWI had a higher risk of OAB, indicating a positive correlation between the two (Figure 2). The smooth curve fitting revealed a non-linear relationship between OAB and other obesity markers, including WC and BMI. Next, we used a segmented regression model to fit each interval and calculate threshold effects (Table 4). In addition, to compare the odds ratios of WWI with other obesity markers, we calculated their *z*-scores and used them in a linear model. Compared to other obesity markers including WC and BMI, WWI showed a stronger correlation with the risk of OAB (WWI: OR = 1.42; BMI: OR = 1.27; WC: OR = 1.32). This suggests that WWI may be a better predictor of the risk of OAB compared to other obesity markers.

**Table 4:** Threshold effect analysis of WWI, BMI, and WC on OAB; associations between *z*-scores of obesity markers and OAB risk.

	WWI	BMI	WC
Fitting by standard linear model			
OR (95%CI) *P*-value	1.41 (1.02, 1.74) *P* < 0.01	1.05 (1.02–1.32) *P* < 0.01	1.12 (1.04–1.39) *P* < 0.01
Fitting by two-piecewise linear model			
Inflection point (K)			
>K	1.34 (1.19–1.54) <0.01	1.20 (1.15–1.46) <0.01	1.08 (0.87–1.32) 0.52
<K	0.76 (0.56–1.23) 0.32	1.02 (0.76–1.14) 0.13	1.22 (1.03–1.32) <0.01
Likelihood ratio	0.08	0.13	0.32
*z*-scores (continuous)	1.42 (1.13–1.53) <0.01	1.27 (1.08–1.47) <0.01	1.32 (1.12–1.43) <0.01

## 4 Discussion

This research investigated the relationship between WC and OAB among Americans. In this cross-sectional study involving 35,950 subjects, we found that individuals with a higher WWI have a higher risk of OAB. Subgroup analysis and interaction testing showed that the relationship between WWI and OAB is consistent across various population characteristics. Furthermore, we found that the association between WWI and OAB is stronger than that of other obesity-related indicators, indicating that WWI may be a more accurate predictor of OAB than other obesity-related indicators. Our research suggests that WWI may be able to predict the incidence of OAB and that WWI-based obesity management may help to reduce the risk of OAB.

The OAB is a common urological disease, which not only significantly affect the patient’s quality of life but also lead to economic losses and higher medical costs. With the increasing pressure of society and changes in lifestyle, the prevalence of OAB has increased over the years. Although the anatomy and pathophysiology of OAB symptoms are significantly different in men and women, there may be some common features of the potential bladder pathophysiology. Changes in bladder function in both men and women may be related to changes in the vascular system, epithelial cells, neurons, smooth muscle, and connective tissues, resulting in OAB (20). In elderly women, the results of the lower urinary tract symptoms questionnaire show similar results to those of age-matched men (21). A systematic review conducted by Bunn et al. showed that there is increasing evidence to suggest a correlation between OAB and metabolic syndrome (METS) (7). METS patients are at a greater risk of developing OAB due to various factors. These include excessive sympathetic nervous activity, pro-inflammatory states, oxidative stress, and impaired endothelial function, as well as ischemic damage to the bladder wall during storage, decreased bladder and bladder neck perfusion, increased non-esterified fatty acids, and decreased nitric oxide. OAB may be caused by various pathological changes in the bladder wall, including alterations in smooth muscle or neural mechanisms. Insulin resistance caused by obesity is a significant component of METS. Uzun et al. (22) found that the serum insulin level was higher in patients with OAB relative to controls. Additionally, the homeostasis model assessment of insulin resistance (HOMA-IR) index, which is used to measure insulin resistance, was significantly higher in the OAB group compared to the control group. High-density lipoprotein cholesterol levels were significantly lower in females with OAB.

Currently, many studies have confirmed that obesity is a risk factor for urinary incontinence (23, 24). Multiple anthropometric measurements, such as BMI and WC, have been used as measures of obesity. Studies investigating the relationship between obesity and OAB are heterogeneous in their results. Some studies (9–12) have shown a positive association between higher BMI and OAB, while other studies (13, 14) have not shown a significant correlation. WC is a clinical parameter that is used to indirectly assess visceral fat accumulation. Recent studies have shown a significant positive association between WC increase and the risk of OAB (25). A large number of studies have proposed the “obesity paradox” phenomenon of BMI, which states that participants who are overweight or obese tend to have better outcomes, particularly in patients with coronary artery disease and acute myocardial infarction (15, 17, 26). When WC was applied to a comparative study focused on heart failure, similar phenomena were also observed (27, 28). This unexpected paradox may be due to the inability of BMI and WC to distinguish between muscle mass and fat mass. WWI is a new developed obesity measurement that can more accurately predict whole-body fat percentage than the traditional BMI-based equation (15, 29). Recent research has confirmed the ability of WWI to distinguish between muscle mass and fat mass, and its application has extended to various fields, including cardiovascular disease and obesity (30). Therefore, we applied WWI in this cross-sectional study to evaluate the extent of “true obesity.” To the best of our knowledge, this is the first study to evaluate the relationship between WWI and OAB, highlighting the increased risk of OAB associated with higher WWI levels.

Currently, the mechanisms linking WWI and OAB are not well understood. It is possible that multiple mechanisms may be involved. Firstly, an increase in WWI may be caused by mechanical factors leading to increased intra-abdominal and bladder pressure. Additionally, the presence of the secreted leptin and inflammatory cytokines produced by visceral adipose tissue may lead to noradrenergic sympathetic activity and urothelial irritation (31). Several studies have already confirmed the significant correlation between abdominal fat accumulation and metabolic syndrome, which is believed to be related to chronic pelvic ischemia and urothelial dysfunction (8, 32, 33).

This study also has some limitations. Firstly, due to the cross-sectional design of the study, it provides evidence for a positive relationship between higher WWI levels and a higher risk of OAB. However, the causal relationship between the two variables cannot be explained. Secondly, OAB is a conglomeration of symptoms caused by multiple factors. Despite controlling for some relevant confounding factors, we cannot completely eliminate the influence of other confounding variables. Therefore, our results should be treated with caution. Thirdly, given that our findings are based on a single country, we cannot determine whether they are applicable to other countries.

Despite these limitations, our research still has some advantages. Firstly, our study takes into account the sample weights and uses data from a nationwide sample, which makes our results more representative of the overall population in the United States. Secondly, as the sample size is large, after correcting for confounding factors, we used subgroup analysis to confirm the robustness of our regression analysis. Finally, we further investigated the relationship between WWI and OAB, finding that it is more significant than the relationship between BMI or WC and OAB.

## 5 Conclusion

In this study, we found a positive relationship between higher WWI levels and an increased risk of OAB. Furthermore, the relationship between WWI and OAB is stronger than the correlation between other obesity markers and OAB, indicating that WWI may serve as a simple anthropometric indicator for predicting OAB.

## Data Availability

The original contributions presented in the study are included in the article/Supplementary material, further inquiries can be directed to the corresponding authors.
