# Supplementary material for: Association between weight-adjusted waist index and overactive bladder: a cross-sectional study based on 2009–2018 NHANES
Source: Front Nutr. 2024 Sep 4;11:1423148. doi: 10.3389/fnut.2024.1423148 (PMC11408301; doi:10.3389/fnut.2024.1423148)
Supplement: Supplementary file 1 [file Table_1.docx]

Table s1. Criteria for conversion of symptom frequencies recorded in NHANES to OABSS scores

| Urge urinary incontinence frequency  (from NHANES) | Urge urinary incontinence score  (according to OABSS) |
| --- | --- |
| Never | 0 |
| Less than once a month | 1 |
| A few times a month | 1 |
| A few times a week | 2 |
| Every day and/or night | 3 |
| Nocturia frequency(from NHANES) | Nocturia score (according to OABSS) |
| 0 | 0 |
| 1 | 1 |
| 2 | 2 |
| 3 | 3 |
| 4 | 3 |
| 5 or more | 3 |

NHANES = National Health and Nutrition Examination Survey; OABSS = Overactive Bladder Symptom Score
